# Regulation of the Spontaneous Augmentation of Na_V_1.9 in Mouse Dorsal Root Ganglion Neurons: Effect of PKA and PKC Pathways

**DOI:** 10.3390/md8030728

**Published:** 2010-03-19

**Authors:** Jun-ichi Kakimura, Taixing Zheng, Noriko Uryu, Nobukuni Ogata

**Affiliations:** 1 Technical Center, Hiroshima University, Hiroshima, 734-8551, Japan; 2 Department of Neurophysiology, Graduate School of Biomedical Sciences, Hiroshima University, Hiroshima, 734-8551, Japan; E-Mails: tei0322@hiroshima-u.ac.jp (T.Z.); uryu@asahi.ac.jp (N.U.); ogatan@hiroshima-u.ac.jp (N.O.)

**Keywords:** Na^+^ channel, tetrodotoxin, dorsal root ganglion, patch clamp, PKA, PKC

## Abstract

Sensory neurons in the dorsal root ganglion express two kinds of tetrodotoxin resistant (TTX-R) isoforms of voltage-gated sodium channels, Na_V_1.8 and Na_V_1.9. These isoforms play key roles in the pathophysiology of chronic pain. Of special interest is Na_V_1.9: our previous studies revealed a unique property of the Na_V_1.9 current, *i.e.*, the Na_V_1.9 current shows a gradual and notable up-regulation of the peak amplitude during recording (“spontaneous augmentation of Na_V_1.9”). However, the mechanism underlying the spontaneous augmentation of Na_V_1.9 is still unclear. In this study, we examined the effects of protein kinases A and C (PKA and PKC), on the spontaneous augmentation of Na_V_1.9. The spontaneous augmentation of the Na_V_1.9 current was significantly suppressed by activation of PKA, whereas activation of PKA did not affect the voltage dependence of inactivation for the Na_V_1.9 current. On the contrary, the finding that activation of PKC can affect the voltage dependence of inactivation for Na_V_1.9 in the perforated patch recordings, where the augmentation does not occur, suggests that the effects of PMA are independent of the augmentation process. These results indicate that the spontaneous augmentation of Na_V_1.9 was regulated directly by PKA, and indirectly by PKC.

## 1. Introduction

Voltage-gated sodium channels (Na^+^ channels) mediate the transient increase in Na^+^ conductance that underlies the action potential of neurons and other excitable cells. In mammals, a family of Na^+^ channel α subunits (designated Na_V_1.1–Na_V_1.9) exhibit unique patterns of anatomical expression and varied functional and pharmacological properties [1,2].

Unmyelinated C-fibers originate from small primary afferent neurons of the dorsal root ganglion (DRG), and transmit nociceptive information to the central nervous system. Na^+^ currents expressed in small DRG neurons can be classified into two categories on the basis of their relative sensitivity to tetrodotoxin (TTX); the first, TTX-sensitive (TTX-S) Na^+^ currents and the second, TTX-resistant (TTX-R) Na^+^ currents. TTX-R Na^+^ currents can be further subdivided into the current mediated by Na_V_1.8, known as SNS [3–6], and the current mediated by Na_V_1.9, known as NaN [7–11].

The Na_V_1.9 current is characterized by a low activation threshold of about −60 mV and the depolarized steady-state inactivation (*h*_∞_) curve [7,11–13]. From these observations, the Na_V_1.9 current may mainly regulate subthreshold excitability of small DRG neurons [7,10,11,13]. Our previous studies have demonstrated gradual and notable increase-decrease of the peak amplitude of the Na_V_1.9 current during whole-cell patch clamp recording (referred to as ‘spontaneous augmentation of Na_V_1.9’). The spontaneous augmentation of Na_V_1.9 was not observed in the presence intracellular ATP (3 mM) and by using nystatin-perforated patch clamp recording. These results suggest possible involvement of the intracellular environment in the spontaneous augmentation of Na_V_1.9 [11].

It is well known that protein kinases A and C (PKA and PKC), and phosphatases, as well as other proteins such as Ca^2+^-calmodulin-dependent kinase, growth-factor-dependent receptor tyrosine kinases, extracellular signal-regulated kinases and heterotrimeric G proteins, also modulate Na^+^ channels [14–16]. At present, endogenous factors that regulate the spontaneous augmentation of Na_V_1.9 have not been identified. In sensory neurons, activation of PKA and PKC affects the biophysical properties of Na^+^ channels [1]. As a first step to the above issue, we focused on the possible involvement of the PKA and PKC pathways in the spontaneous augmentation of Na_V_1.9. For this purpose, in this study we examined the effects of activators and inhibitors of PKA and PKC on the spontaneous augmentation of Na_V_1.9, using Na_V_1.8 knock-out (KO) mice [5,11,13] to record the Na_V_1.9 current in isolation.

## 2. Results and Discussion

### 2.1. Characterization of the Na_V_1.9 current

**Figure 1:**
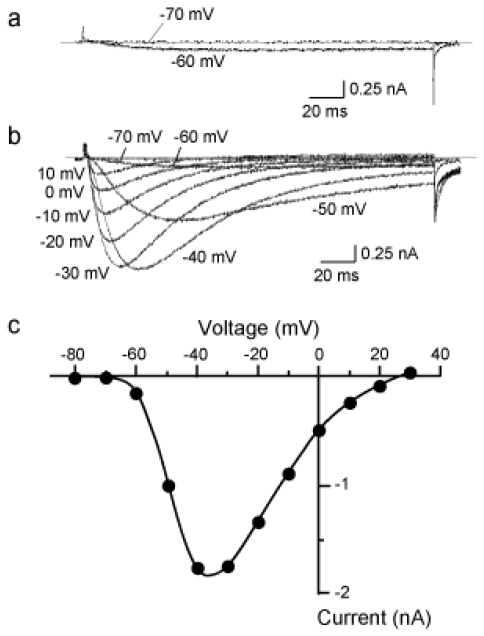
Typical examples of the tetrodotoxin-resistant (TTX-R) Na^+^ current mediated by Na_V_1.9. The Na_V_1.9 current was recorded from the small (diameter < 25 μm) dorsal root ganglion (DRG) neuron prepared from the Na_V_1.8 knock-out (KO) mouse. Currents were elicited by 200 ms test pulse (*V*_T_) from a holding potential (*V*_H_) of −80 mV. (a) The activation threshold for the Na_V_1.9 current. The Na_V_1.9 current could be activated at −60 mV, whereas *V*_T_ to −70 mV failed to elicit. (b) A family of superimposed Na_V_1.9 currents evoked by *V*_T_s between −70 and +10 mV in 10-mV increments from *V*_H_ of −80 mV. The external solution contained 200 nM TTX. Voltage labels attached to traces indicate *V*_T_s. (c) The current-voltage curve for the Na_V_1.9 current shown in panel (b). Peak amplitudes of the currents were plotted against *V*_T_.

The detailed properties of the Na_V_1.9 current was already described in our previous reports [11,13,17]. A typical example of the Na_V_1.9 currents is shown in Figure 1. Currents were evoked by a 200 ms test pulse (*V*_T_) from a holding potential (*V*_H_) of −80 mV. TTX-sensitive (TTX-S) Na^+^ currents were completely eliminated by 200 nM TTX contained in the external solution. The Na_V_1.9 current had an activation threshold at about −60 mV (Figure 1a), which was more negative than thresholds for Na^+^ currents mediated by Na_V_1.8 and other TTX-S Na^+^ currents (about −40 mV) [13]. Time courses of activation and inactivation of the Na_V_1.9 current were extremely prolonged, particularly at lower activation voltages (Figure 1b). Figure 1c illustrates the current-voltage relationship for the currents shown in Figure 1b.

### 2.2. Modification of the spontaneous augmentation of Na_V_1.9 by PKA and PKC

**Figure 2:**
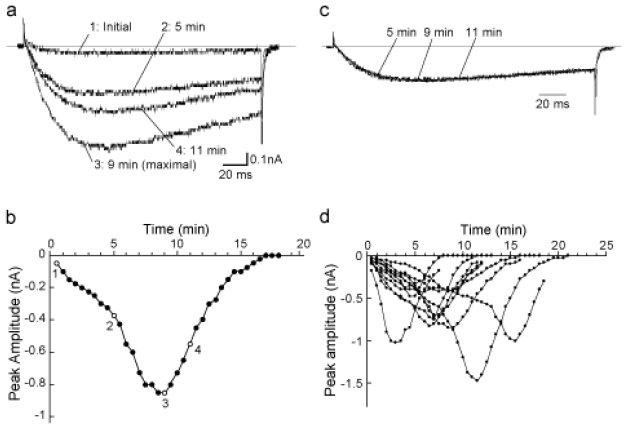
(a) Time courses of the Na_V_1.9 currents recorded with normal pipette solution. The Na_V_1.9 current was evoked by *V*_T_ to −10 mV from *V*_H_ of −80 mV, and command pulses were applied every 30 s. Then, selected traces were superimposed. (b) Typical example of the spontaneous augmentation of Na_V_1.9. Peak amplitudes of the Na_V_1.9 current were plotted as a function of time after onset of the spontaneous augmentation of Na_V_1.9. Numbers indicate the traces shown in panel (a). (c) Traces of the Na_V_1.9 currents shown in panel (a) were normalized and then superimposed. (d) Time courses of the Na_V_1.9 currents in all cases examined.

As previously described [11,17], the peak amplitude of the Na_V_1.9 current remarkably increased during conventional whole-cell recording with control pipette solution. As shown in Figure 2, the peak amplitude of the Na_V_1.9 current gradually increased up to 18.7 ± 5.0-fold of the initial value (n = 10), and then diminished towards the initial value. The total duration of the spontaneous augmentation of Na_V_1.9 was 12.4 ± 1.7 min (n = 10). In addition, activation and inactivation kinetics of this current did not change during the spontaneous augmentation of Na_V_1.9 (Figure 2c).

**Figure 3:**
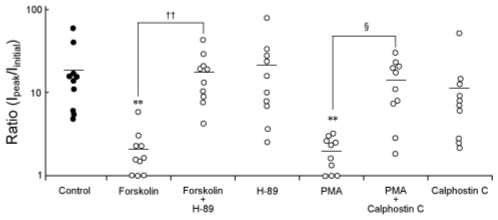
Modification of the spontaneous augmentation of Na_V_1.9 by addition of the PKA activator forskolin and the PKC activator PMA to the pipette solution. The ordinate shows the ratio of maximal amplitude during recording to the initial amplitude of the Na_V_1.9 current (*I*_maximal_/*I*_initial_). A ratio > 1 indicates the occurrence of the spontaneous augmentation of Na_V_1.9, whereas a ratio = 1 indicates no change or reduction of the peak amplitude of the Na_V_1.9 current. The plots are shown on a logarithmic scale. Circles indicate the ratios obtained for individual trials. Filled circles, control; open circles, treated groups. Horizontal bars indicate the mean values of each group. **P < 0.01 *versus* control, ††*P* < 0.01 *versus* forskolin, and §*P* < 0.05 *versus* PMA.

Figure 3 summarizes the intensity changes of the spontaneous augmentation of Na_V_1.9, which were induced by compounds added to the pipette solution. In the presence of a PKA activator, forskolin (10 μM), the intensity of the spontaneous augmentation of Na_V_1.9 was significantly attenuated (2.1 ± 0.5-fold: n = 10). Although a PKA inhibitor, H-89 (10 μM). alone had no detectable effect on the intensity of the spontaneous augmentation of Na_V_1.9 (21.1 ± 7.3-fold: n = 10), the effect of forskolin was significantly inhibited by treatment with H-89 (17.3 ± 3.5-fold: n = 10). A PKC activator, PMA (100 nM), had significant suppressive effect on the intensity of the augmentation of Na_V_1.9 (2.0 ± 0.3-fold: n = 10). In the presence of a PKC inhibitor, calphostin C (100 nM), the effect of PMA on the augmentation of Na_V_1.9 was significantly inhibited (14.2 ± 2.8-fold: n = 10). Similarly to H-89, calphostin C alone had no discernable effect on the spontaneous augmentation of Na_V_1.9 (11.3 ± 4.4-fold: n = 10).

### 2.3. Possible involvement of PKA and PKC pathways in the voltage dependence of inactivation for the Na_V_1.9 current

**Figure 4:**
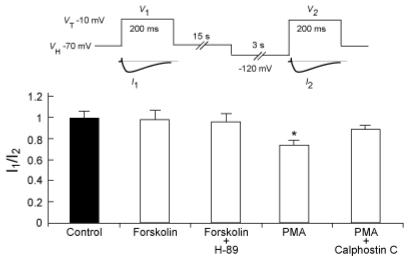
The change of the voltage-dependence of inactivation for the Na_V_1.9 current by activation of PKC. The Na_V_1.9 currents were evoked by the pulse protocol shown in the diagram. We previously confirmed that the duration of *V*_H_ for 3 s was sufficient to achieve steady state availability for Na_V_1.9 [11]. Peak amplitudes of the Na_V_1.9 currents (*I*_1_ and *I*_2_) were monitored for 10 min after commencing recording, and the resulting *I*_1_/*I*_2_ ratios were compared. *P < 0.05 *versus* control.

We next examined the effects of forskolin and PMA on the voltage dependence of inactivation for the Na_V_1.9 current (Figure 4). The voltage dependence of inactivation was determined by measuring peak amplitudes of the Na_V_1.9 currents evoked by *V*_T_ to −10 mV from *V*_H_s of −70 mV (*I*_1_) and −120 mV (*I*_2_) intermittently (every 1 minute), and then the ratio of *I*_1_/*I*_2_ obtained from each group was compared. Just after commencing recording, there were no significant differences between each group (data not shown). 10 minutes after commencing recording, the presence of forskolin (10 μM) alone, or the co-presence of forskolin and H-89 (10 μM), did not significantly change the ratios of *I*_1_/*I*_2_ (1.00 ± 0.08: n = 8, and 0.99 ± 0.06: n = 6, respectively) *versus* control (0.99 ± 0.07: n = 8). On the contrary, 100 nM PMA significantly reduced the ratio of *I*_1_/*I*_2_ (0.75 ± 0.07: n = 8) as compared to control. Calphostin C (100 nM) inhibited PMA-induced reduction of *I*_1_/*I*_2_ (0.91 ± 0.03: n = 5).

### 2.4. The effect of PMA on the h_∞_ curve for the Na_V_1.9 current is reproducible under nystatin-perforated patch clamp recording

**Figure 5:**
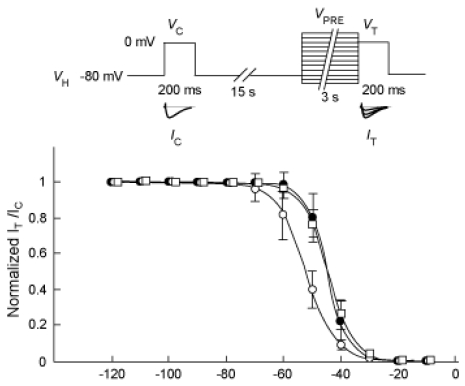
The *h*_∞_ curve for the Na_V_1.9 current under nystatin-perforated patch clamp recording. The diagram illustrates the pulse protocol. Two identical pulses to 0 mV for 200 ms were applied 15 s prior (*V*_C_) and immediately subsequent (*V*_T_) to the conditioning pre-pulse (*V*_PRE_) of −120 to 10 mV for 3 s from *V*_H_ of −80 mV. *V*_C_ was delivered to minimize the error arising from possible fluctuation of the current (see experimental section). The pulse protocol was applied every 40 s. The peak amplitude of the current (*I*_T_) evoked by *V*_T_ was divided by the peak amplitude of the current (*I*_C_) evoked by *V*_C_. The ratio of *I*_T_*/I*_C_ was normalized and plotted against *V*_PRE_. The *h*_∞_ curve for Na_V_1.9 current was determined, before (control, filled circle) and 10 min after bath application of PMA (open circle) or forskolin (open square).

The spontaneous augmentation of Na_V_1.9 was prevented by using nystatin-perforated patch clamp recording [11]. Therefore, we further examined the effect of forskolin and PMA on the *h*_∞_ (voltage dependence of steady-state inactivation) curve for the Na_V_1.9 current in such a situation. The Na_V_1.9 current was evoked by *V*_T_ to 0 mV after various *V*_PRE_s steps ranging from −120 mV to 10 mV in 10-mV steps for 3 s (see diagram of Figure 5). Figure 5 shows *h*_∞_ curves for the Na_V_1.9 current before (control) and after application of 100 nM PMA or 10 μM forskolin for 10 min. The *h*_∞_ curves were fitted to the Boltzmann equation:

(1) $$I/I_{\max}=1/\{1+\exp[(V_{\text{PRE}}-V_{1/2})/k]\}$$

Where *I* is the peak amplitude of the Na_V_1.9 current obtained by *V*_T_, *I*_max_ is the peak amplitude of the Na_V_1.9 current evoked by *V*_C_, *V*_1/2_ is the half-maximum inactivation voltage, and *k* is the slope factor. We found significant differences (*P* < 0.05) between parameters obtained after PMA application (n = 5, *V*_1/2_ = −52.1 ± 5.4 mV, *k* = 5.17 ± 0.44 mV) *versus* control (n = 4, *V*_1/2_ = −44.7 ± 6.4 mV, *k* = 3.75 ± 0.15 mV). On the contrary, there was no significant change between values obtained after forskolin application (n = 5, *V*_1/2_ = −44.6 ± 2.1 mV, *k* = 4.44 ± 0.51 mV) from the control.

### 2.5. Discussion

The expression of the Na_V_1.9 current is confined to the subpopulation of primary afferent neurons with a small cell-body diameter similarly to the Na_V_1.8 current [4–8,10,11,18]. This observation suggests that Na_V_1.9 plays an important role in nociception, similar to Na_V_1.8. On the other hand, the electrophysiological properties of the Na_V_1.9 current differ notably from those of the Na_V_1.8 current, *i.e.*, the Na_V_1.9 current has a more hyperpolarized activation threshold, much slower activation and inactivation kinetics, and a more depolarized *h*_∞_ curve [11,13]. These observations suggest that the role of Na_V_1.9 in nociception may be distinct from that of Na_V_1.8.

It was reported that the sustained membrane depolarization (over several hundreds of milliseconds), which is resistant to TTX, is observed in myenteric ganglia [12]. In addition, we previously observed similar sustained membrane depolarization mediated by Na_V_1.9 in small DRG neurons [13]. The Na_V_1.9 current may have facilitatory functions in the generation of action potential. On the other hand, *h*_∞_ curves for other Na^+^ currents are more depolarized than that of Na_V_1.9 [11,13]. Taking into consideration these properties, sustained activation of Na_V_1.9 may decrease the availability of other Na^+^ channels, and then conversely act to inhibit generation of action potential. Thus, the regulation of membrane potential by Na_V_1.9 may be facilitatory or inhibitory to generating an action potential, in a context-dependent manner.

In addition, we observed a sporadic and remarkable increase of the peak amplitude of the Na_V_1.9 current followed by decrease of the peak amplitude during whole-cell patch clamp recording (“spontaneous augmentation of Na_V_1.9”) [11]. We previously reported that the spontaneous augmentation of Na_V_1.9 was inhibited by recording with the nystatin-perforated patch clamp technique, and in the presence of intracellular ATP [11,17]. These results suggest a possible involvement of the intracellular environment in the spontaneous augmentation of Na_V_1.9.

Analyses of brain Na^+^ channels have shown that the cytoplasmic loop between domains DI and DII of Na^+^ channels possesses several shared PKA and PKC phosphorylation sites [19–20]. In addition, the inactivation gate has a unique site for PKC phosphorylation [22,23]. In fact, activation of PKA reduces the peak amplitude of brain Na^+^ channels without the shift of the steady-state properties [20,24]. On the other hand, activation of PKC has regulatory effects on skeletal muscle Na^+^ channels [25], brain Na^+^ channels [26] and peripheral nerve Na^+^ channel expressed in *Xenopus* oocytes [27]. However, the effects of PKA and PKC on the spontaneous augmentation of Na_V_1.9 in sensory neurons have not been investigated.

First, we focused on the effect of PKA on the spontaneous augmentation of Na_V_1.9. The spontaneous augmentation of Na_V_1.9 was significantly suppressed in the presence of forskolin, and H-89 significantly inhibited this suppressive effect of forskolin (Figure 3). These results indicate that the spontaneous augmentation of Na_V_1.9 is suppressed by activation of PKA. It is well known that the amplitude of Na^+^ current is strongly affected by steady-state inactivation of the channel. However, forskolin did not change the *h*_∞_ curve parameters for the Na_V_1.9 current in comparison with the control (Figures 4 and 5). This suggests that inhibition of the spontaneous augmentation of Na_V_1.9 by PKA activation was not due to the hyperpolarizing shift of the *h*_∞_ curve for the Na_V_1.9 current.

Next, we focused on the effect of PKC on the spontaneous augmentation of Na_V_1.9. PMA significantly reduced the intensity of augmentation, and the effect of PMA was significantly suppressed by Calphostin C (Figure 3). However, application of PMA induced the hyperpolarizing shift of the voltage dependence of inactivation for the Na_V_1.9 current. Thus, apparent suppression of the spontaneous augmentation of Na_V_1.9 by PMA was largely due to hyperpolarizing shift of the *h*_∞_ curve for the Na_V_1.9 current.

A similar phenomenon has also been reported by Baker *et al.,* who showed that intracellular GTP [28] or activation of PKC [29] induces the up-regulation of the Na_V_1.9 current. There are distinct dissimilarities between the up-regulation reported by Baker *et al.* and the spontaneous augmentation of Na_V_1.9 described in the present study and also in our previous reports [11,13,17]. Namely, (1) the up-regulation reported by Baker *et al.* was observed in the presence of intracellular ATP. On the contrary, the spontaneous augmentation of Na_V_1.9 in our experiments occurred only in the absence of intracellular ATP. (2) the up-regulation reported by *Baker et al.* was mediated by intracellular GTP or activation of PKC. On the other hand, we find that intracellular GTP has no effect on the spontaneous augmentation of Na_V_1.9 (unpublished data). (3) *Baker et al.* showed only an increment of peak amplitude of the Na_V_1.9 current, and did not show the entire time course of the phenomenon [28,29]. The spontaneous augmentation of Na_V_1.9 was composed of an increase and a subsequent decrease of peak amplitude of the Na_V_1.9 current. From these observations, the up-regulation reported by *Baker et al.* may be distinct from the spontaneous augmentation of Na_V_1.9 in our studies.

A recent behavioral study showed that the PKA inhibitor, H-89, suppresses bee venom-induced mechanical hyperalgesia in rats [30], and the activation of PKA is conductive to the inflammatory mechanical hyperalgesia [31,32]. In addition, activity of specific PKC isozymes is increased in inflammatory-pain models in rats [33–36]. From these observations, the Na_V_1.9 channel may be regulated not to increase the amplitude of the Na_V_1.9 current, *i.e.*, the spontaneous augmentation of Na_V_1.9 under pathological condition by PKA and/or PKC activation.

Recently, it has been reported that Na_V_1.9 underlies nociceptive behavior after peripheral inflammation in Na_V_1.9 KO mice [37]. In addition, histochemical study on Na_V_1.9 channel proteins in axons of normal and complete Freund’s adjuvant-inflamed rats showed significant decrease of the proportion of Na_V_1.9-labeled unmyelinated axons, and no change in the proportion of labeled myelinated axons following inflammation [18]. Taken together, the Na_V_1.9 current contributes to pain signaling, and may alter under pathophysiological conditions.

## 3. Experimental Section

### 3.1. Isolation of single DRG neurons and cell culture

The protocols were approved by Hiroshima University Animal Ethics Committee. Dissociation of single DRG neurons and their culture was described previously. [11,13]. Briefly, adult mice were sacrificed by cervical dislocation under ethylcarbamate anesthesia (3 mg/g; Wako Pure Chemicals, Osaka, Japan). DRGs from all segments of the spinal cord were dissected from Na_V_1.8 KO mice [5] and desheathed in ice-cold Ca^2+^/Mg^2+^-free phosphate-buffered saline (PBS(−)). The isolated DRGs were incubated sequentially in PBS(−) containing 0.2% collagenase (Wako Pure Chemicals) and 0.1% trypsin (Sigma, St. Louis, Mo., USA), each for 20 min at 37 °C. DRGs were then dissociated by trituration with fire-polished Pasteur pipettes in culture medium composed of Dulbecco’s modified Eagle medium 10% (vol./vol.) heat-inactivated fetal calf serum.

The dissociated cells were plated on 35 mm plastic tissue-culture dishes pre-coated with 0.01% poly-l-lysine (Sigma) and maintained in culture medium supplemented with penicillin (100 IU/mL) and streptomycin (100 μg/mL). All cultured cells were maintained at 37 °C in 5% CO_2_/95% air. Cells were used for experiments after short-term culture (4–12 h after plating). At this time in culture, neurite outgrowth was not observed. We defined DRG neurons that were smaller than 25 μm in diameter as small neurons [13,28], and small neurons thus defined were used throughout the study.

### 3.2. Electrophysiology

Voltage clamp recordings were performed using an Axopatch 200A amplifier (Axon Instruments, Union City, CA, USA). The Na^+^ currents were recorded by using either the conventional whole-cell patch clamp technique [38] or the nystatin-perforated patch clamp technique [39] at room temperature (22–24 °C). Data were low-pass-filtered at 5 kHz with a four-pole Bessel filter and sampled digitally at 25–100 kHz. In some experiments, capacitive and leakage currents were subtracted digitally using the P-P/4 procedure [4].

The pipette (internal) solution contained 10 mM NaCl, 110 mM CsCl, 20 mM tetra-ethylammonium (TEA)-Cl, 2.5 mM MgCl_2_, 5 mM 4-2-hydroxylethyl-1-piperazine-ethanesulfonic acid (HEPES) and 5 mM ethylene glycol tetraacetic acid (EGTA). The pH of the pipette solution was adjusted to 7.0 with CsOH. Osmolarity was adjusted to 290 mosmol/kg with glucose. A part of the experiments were performed with the pipette solution containing phorbol 12-myristate 13-acetate (PMA), calphostin C, forskolin and H-89 (Sigma). For the pipette solution used in nystatin-perforated patch recordings, a stock solution containing 10mg/mL nystatin (Wako) was prepared and added to the pipette solution to final concentration of 500 μg/mL. Patch clamp pipettes were made from borosilicate capillary by using a PP-83 puller (Narishige, Tokyo, Japan) and heat-polished with MF-83 microforge (Narishige). The DC resistance of patch electrodes was 1–1.5 MΩ for the conventional whole-cell patch and 3–5 MΩ for the nystatin-perforated patch, respectively.

The external solution contained 100 mM NaCl, 30 mM TEA-Cl, 5 mM CsCl, 1.8 mM CaCl_2_, 1 mM MgCl_2_, 0.1 mM CdCl_2_, 5 mM HEPES, 25 mM glucose, 5 mM 4-aminopyrimidine (4-AP). The pH of the external solution was adjusted to 7.4 with HCl. TEA-Cl and 4-AP were added to abolish K^+^ currents and CdCl_2_ was added to abolish Ca^2+^ currents [4]. In addition, TTX (Sanko, Tokyo, Japan) was added to eliminate TTX-S Na^+^ currents. Osmolarity was adjusted to 290 mosmol/kg with glucose. A part of the experiments were performed with the external solution containing PMA and forskolin (Sigma). Liquid junction potentials between pipette and external solutions were compensated by adjusting the zero current potential to the liquid junction potential. Only cells showing an adequate voltage and space clamp [11] were used.

When measuring ion channel kinetics, it generally take a considerable time to execute the protocol, since a sufficient recovery period for the channel must be allowed between each test pulse (*V*_T_). For various reasons, the amplitudes of Na^+^ currents are not always constant during recording, e.g., due to run-down of the current or to instability of the seal condition. This can be checked by applying a control pulse (*V*_C_) before each *V*_T_. To avoid a possible time-dependent fluctuation of the analysis, *V*_C_ to a fixed voltage as applied 15 s before *V*_T_ or conditioning pre-pulse (*V*_PRE_). The amplitude of the current (*I*_C_) evoked by *V*_C_ served as a calibrator. The results are given as mean ± standard error of the mean (S.E.M.). Statistical significance of differences was determined using Wilcoxon *t*-test and Mann-Whitney *U*-test. Differences were considered significant if *P* < 0.05.

## 4. Conclusions

The spontaneous augmentation of the Na_V_1.9 current was significantly suppressed by activation of PKA, whereas activation of PKA did not affect the voltage dependence of inactivation for the Na_V_1.9 current. On the contrary, the finding that activation of PKC can affect the voltage dependence of inactivation for Na_V_1.9 in the perforated patch recordings, where the augmentation does not occur, suggests that the effects of PMA are independent of the augmentation process. These results indicate that the spontaneous augmentation of Na_V_1.9 was regulated directly by PKA, and indirectly by PKC.
